# A Review of Feature Selection and Feature Extraction Methods Applied on Microarray Data

**DOI:** 10.1155/2015/198363

**Published:** 2015-06-11

**Authors:** Zena M. Hira, Duncan F. Gillies

**Affiliations:** Department of Computing, Imperial College London, London SW7 2AZ, UK

## Abstract

We summarise various ways of performing dimensionality reduction on high-dimensional microarray data. Many different feature selection and feature extraction methods exist and they are being widely used. All these methods aim to remove redundant and irrelevant features so that classification of new instances will be more accurate. A popular source of data is microarrays, a biological platform for gathering gene expressions. Analysing microarrays can be difficult due to the size of the data they provide. In addition the complicated relations among the different genes make analysis more difficult and removing excess features can improve the quality of the results. We present some of the most popular methods for selecting significant features and provide a comparison between them. Their advantages and disadvantages are outlined in order to provide a clearer idea of when to use each one of them for saving computational time and resources.

## 1. Introduction

In machine learning as the dimensionality of the data rises, the amount of data required to provide a reliable analysis grows exponentially. Bellman referred to this phenomenon as the “curse of dimensionality” when considering problems in dynamic optimisation [1]. A popular approach to this problem of high-dimensional datasets is to search for a projection of the data onto a smaller number of variables (or features) which preserves the information as much as possible. Microarray data is typical of this type of small sample problem. Each data point (sample) can have up to 450,000 variables (gene probes) and processing a large number of data points involves high computational cost [2]. When the dimensionality of a dataset grows significantly there is an increasing difficulty in proving the result statistically significant due to the sparsity of the meaningful data in the dataset in question. Large datasets with the so-called “large *p*, small *n*” problem (where *p* is the number of features and *n* is the number of samples) tend to be prone to overfitting. An overfitted model can mistake small fluctuations for important variance in the data which can lead to classification errors. This difficulty can also increase due to noisy features. Noise in a dataset is defined as “*the error in the variance of a measured variable*” which can result from errors in measurements or natural variation [3]. Machine learning algorithms tend to be affected by noisy data. Noise should be reduced as much as possible in order to avoid unnecessary complexity in the inferred models and improve the efficiency of the algorithm [4]. Common noise can be divided into two types [5]:

1. Attribute noise.
2. Class noise.

Attribute noise is caused by errors in the attribute values (wrongly measured variables, missing values) while class noise is caused by samples that are labelled to belong in more than one class and/or misclassifications.

As the dimensionality increases the computational cost also increases, usually exponentially. To overcome this problem it is necessary to find a way to reduce the number of features in consideration. Two techniques are often used:

1. Feature subset selection.
2. Feature extraction.

Cancer is among the leading causes of death worldwide accounting for more than 8 million deaths according to the World Health Organization. It is expected that the deaths from cancer will rise to 14 million in the next two decades. Cancer is not a single disease. There are more than 100 known different types of cancer and probably many more. The term cancer is used to describe the abnormal growth of cells that can, for example, form extra tissue called mass and then attack other organs [6].

Microarray databases are a large source of genetic data, which, upon proper analysis, could enhance our understanding of biology and medicine. Many microarray experiments have been designed to investigate the genetic mechanisms of cancer, and analytical approaches have been applied in order to classify different types of cancer or distinguish between cancerous and noncancerous tissue. In the last ten years, machine learning techniques have been investigated in microarray data analysis. Several approaches have been tried in order to (i) distinguish between cancerous and noncancerous samples, (ii) classify different types of cancer, and (iii) identify subtypes of cancer that may progress aggressively. All these investigations are seeking to generate biologically meaningful interpretations of complex datasets that are sufficiently interesting to drive follow-up experimentation.

This review paper is structured as follows. The next section is about feature selection methods (filters, wrappers, and embedded techniques) applied on microarray cancer data. Then we discuss feature extraction methods (linear and nonlinear) in microarray cancer data and the final section is about using prior knowledge in combination with a feature extraction or feature selection method to improve classification accuracy and algorithmic complexity.

## 2. Feature Subset Selection in Microarray Cancer Data

Feature subset selection works by removing features that are not relevant or are redundant. The subset of features selected should follow the Occam's Razor principle and also give the best performance according to some objective function. In many cases this is an NP-hard (nondeterministic polynomial-time hard) problem [7, 8]. The size of the data to be processed has increased the past 5 years and therefore feature selection has become a requirement before any kind of classification takes place. Unlike feature extraction methods, feature selection techniques do not alter the original representation of the data [9]. One objective for both feature subset selection and feature extraction methods is to avoid overfitting the data in order to make further analysis possible. The simplest is feature selection, in which the number of gene probes in an experiment is reduced by selecting only the most significant according to some criterion such as high levels of activity. Feature selection algorithms are separated into three categories [10, 11]:

- The* filters* which extract features from the data without any learning involved.
- The* wrappers* that use learning techniques to evaluate which features are useful.
- The* embedded techniques* which combine the feature selection step and the classifier construction.

### 2.1. Filters

Filters work without taking the classifier into consideration. This makes them very computationally efficient. They are divided into multivariate and univariate methods. Multivariate methods are able to find relationships among the features, while univariate methods consider each feature separately. Gene ranking is a popular statistical method. The following methods were proposed in order to rank the genes in a dataset based on their significance [12]:

- (Univariate)* Unconditional Mixture Modelling* assumes two different states of the gene on and off and checks whether the underlying binary state of the gene affects the classification using mixture overlap probability.
- (Univariate)* Information Gain Ranking* approximates the conditional distribution *P*(*C*∣*F*), where *C* is the class label and *F* is the feature vector. Information gain is used as a surrogate for the conditional distribution.
- (Multivariate)* Markov Blanket Filtering* finds features that are independent of the class label so that removing them will not affect the accuracy.

In multivariate methods,* pair t-scores* are used for evaluating gene pairs depending on how well they can separate two classes in an attempt to identify genes that work together to provide a better classification [13]. Their results for the gene pair rankings were found to be* “at least as interesting as the single genes found by an independent evaluation.”*

**Figure 1:**
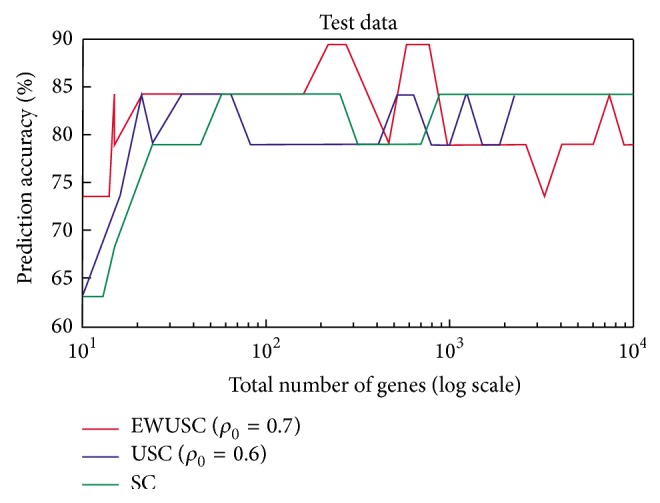
Comparison between EWUSC, USC, and SC on breast cancer data [14].

Methods based on correlation have also been suggested:

(i) (Multivariate)* Error-Weighted Uncorrelated Shrunken Centroid* (EWUSC): this method is based on the* uncorrelated shrunken centroid* (USC) and* shrunken centroid* (SC). The shrunken centroid is found by dividing the average gene expression for each gene in each class by the standard deviation for that gene in the same class. This way higher weight is given to genes whose expression is the same among different samples in the same class. New samples are assigned to the label with the nearest average pattern (using squared distance). The uncorrelated shrunken centroid approach removes redundant features by finding genes that are highly correlated in the set of genes already found by SC. The EWUSC uses both of these steps and in addition adds error-weights (based on within-class variability) so that noisy genes will be downgraded and redundant genes are removed [14]. A comparison is shown in Figure 1 where the three different methods are tested on a relatively small (25000 genes and 78 samples) breast cancer dataset. The algorithms perform well when the number of relevant genes is less than 1000.
(ii) (Multivariate)* Minimum Redundancy Maximum Relevance* (mRMR): mRMR is a method that maximises the relevancy of genes with the class label while it minimises the redundancy in each class. To do so, it uses several statistical measures.* Mutual Information* (MI) measures the information a random variable can give about another, in particular the gene activity and the class label. The method can be applied to both categorical and continuous variables. For categorical (discrete) variables, MI is used to find genes that are not redundant (minimise redundancy) *W* and are maximally relevant *V* with a target label [15] as shown in (1) and (2), respectively:
  (1) W=1S2∑i,j∈SIi,j,
  (2) V=1S∑i∈SIh,i,
 where *I* is the MI, *i* and *j* are genes, |*S*| is the number of features in *S*, and *h* is a class label.
 For continuous variables the *F-statistic* (ANOVA test or regression analysis to check whether the means of two populations are significantly different) is used to find the maximum relevance between a gene and a class label and then the correlation of the gene pair in that class is measured to minimise redundancy [15] as shown in (3) and (4), respectively:
  (3) V=1S∑i∈SFi,h,
  (4) W=1S2∑i,j∈Sci,j,
 where *F* is the *F*-statistic, *i* and *j* are genes, *h* is a class label, |*S*| is the number of features in *S*, and *c* is the correlation. mRMR can be used in combination with entropy. Normalised mutual information is used to measure the relevance and redundancy of clusters of genes. Then the most relevant genes are combined and LOOCV (leave-one-out cross-validation) is performed to find the accuracy [16]. For continuous variables linear relationships are used instead of mutual information. MRMR methods give lower error accuracies for both categorical and discrete data.
(iii) (Multivariate)* Correlation-based feature selection* (CFS) as stated by Hall [17] follows the principal that “*a good feature subset is one that contains features highly correlated with the class yet uncorrelated with each other.*” CFS evaluates a subset by considering the predictive ability of each one of its features individually and also their degree of redundancy (or correlation). The difference between CFS and other methods is that it provides a “*heuristic merit*” for a feature subset instead of each feature independently [18]. This means that given a function (heuristic), the algorithm can decide on its next moves by selecting the option that maximises the output of this function. Heuristic functions can also be designed to minimise the cost to the goal.

*ReliefF* [19] is also widely used with cancer microarray data. It is a multivariate method that chooses the features that are the most distinguishable among the different classes. It repeatedly draws an instance (sample) and, based on its neighbours, it gives most weight to the features that help discriminate it from the neighbours of a different class [20, 21]. A method using independent logistic regression with two steps was also proposed [22]. The first step is a univariate method in which the genes are ranked according to their Pearson correlation coefficients. The top genes are considered in the second phase, which is stepwise variable selection. This is a conditionally univariate method based on the inclusion (or exclusion) of a single gene at a time, conditioned on the variables already included.

**Figure 2:**
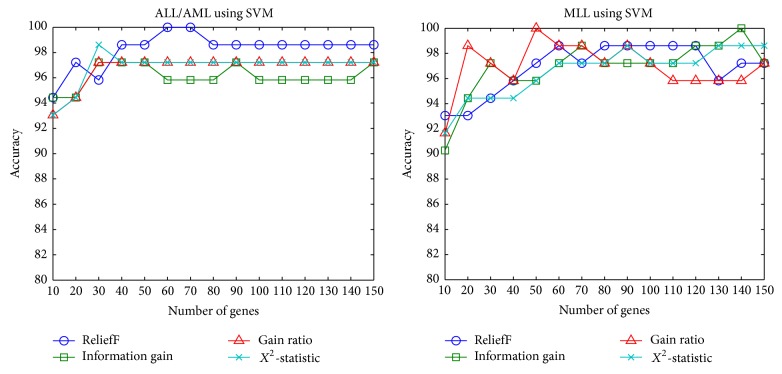
Comparison between ReliefF, Information Gain, Information Gain Ratio, and *X* ^2^ test on ALL and MLL Leukaemia datasets [21].

A comparison of ReliefF,* Information Gain*,* Information Gain Ratio*, and *X* ^2^ is shown in Figure 2. The methods perform similarly across the number of genes selected. Information Gain Ratio is defined as the information gain over the intrinsic information. It performs normalisation to the information gain using split value information. The Pearson *X* ^2^ test evaluates the possibility of a value appearing by chance.

Statistical methods often assume a Gaussian distribution on the data. The central limit theorem can guarantee that large datasets are always normally distributed. Even though all these methods can be highly accurate in classifying information there is no biological significance proven with the genes that are identified by them. None of the above methods have indicated whether the results are actually biologically relevant or not. In addition filter methods are generally faster than wrappers but do not take into account the classifier which can be a disadvantage. Ignoring the specific heuristics and biases of the classifier might lower the classification accuracy.

### 2.2. Wrappers

Wrappers tend to perform better in selecting features since they take the model hypothesis into account by training and testing in the feature space. This leads to the big disadvantage of wrappers, the computational inefficiency which is more apparent as the feature space grows. Unlike filters, they can detect feature dependencies. Wrappers are separated in 2 categories:* Randomised* and* Deterministic*. A comparison is shown in Table 1.

#### 2.2.1. Deterministic Wrappers

A number of deterministic investigations have been used to examine breast cancer such as a combination of a wrapper and* sequential forward selection* (SFS). SFS is a deterministic feature selection method that works by using hill-climbing search to add all possible single-attribute expansions to the current subset and evaluate them. It starts from an empty subset of genes and sequentially selects genes, one at a time, until no further improvement is achieved in the evaluation function. The feature that leads to the best score is added permanently [23]. For classification, support vector machines (SVMs), *k*-nearest neighbours, and probabilistic neural networks were used in an attempt to classify between cancerous and noncancerous breast tumours [24]. Very accurate results were achieved using SVMs. Three methods based on SVMs are very widely used in microarray cancer datasets:

1. *Gradient-based-leave-one-out gene selection* (GLGS) [25–28] was originally introduced for selecting parameters for the SVMs. It starts by applying PCA on the dataset. A vector with scaling factors of the new low-dimensional space is calculated and optimised using a gradient-based algorithm. The pseudo scaling factors of the original genes are calculated. Genes are sequentially selected based on a correlation factor.
2. *Leave-one-out calculation sequential forward selection* (LOOCSFS) is a very widely used feature selection method for cancer data based on sequential forward selection (SFS). It adds features in an initially empty set and calculates the leave-one-out cross-validation error [29]. It is an almost unbiased estimator of the generalisation error using SVMs and C Bound. C Bound is the decision boundary and it is used as a supplementary criterion in the case where different features in the subset have the same leave-one-out cross-validation error (LOOCVE) [26, 30, 31]. SFS can also add constraints [32] on the size of the subset to be selected. It can be used in combination with a recursive support vector machine (R-SVM) algorithm that selects important genes or biomarkers [33]. The* contribution factor*, based on minimal error of the support vector machine, of each gene is calculated and ranked. The top ranked genes are chosen for the subset. LOOCSFS is expected to be an accurate estimator of the generalization error while GLGS scales very well with high-dimensional datasets. The number of the genes in the feature subset for both LOOCSFS and GLGS has to be given in advance which can be a disadvantage since the most important genes are not known in advance. GLGS is said to perform better than LOOCSFS.

#### 2.2.2. Randomised Wrappers

Most randomised wrappers use genetic algorithms (GA) (Algorithm 1) and simulated annealing (Algorithm 2).* Best Incremental Ranked Subset* (BIRS) [35] is an algorithm that scores genes based on their value and class label and then uses incremental ranked usefulness (based on the Markov blanket) to identify redundant genes. Linear discriminant analysis was used in combination with genetic algorithms. Subsets of genes are used as chromosomes and the best 10% of each generation is merged with the previous ones. Part of the chromosome is the discriminant coefficient which indicates the importance of a gene for a class label [36].* Genetic Algorithm-Support Vector Machine* (GA-SVM) [37] creates a population of chromosomes as binary strings that represent the subset of features that are evaluated using SVMs. Simulated annealing works by assuming that some parts of the current solution belong to a better one and therefore proceeds to explore the neighbours seeking for solutions that minimise the objective function and therefore avoid global optima. Hybrid methods with simulated annealing and genetic algorithms have also been used [38]. A genetic algorithm is run as a first step before the simulated annealing in order to get the fittest individuals as inputs to the simulated annealing algorithm. Each solution is evaluated using Fuzzy *C*-Means (a clustering algorithm that uses coefficients to describe how relevant a feature is to a cluster [39, 40]). The problem with genetic algorithms is that the time complexity becomes *O*(*n*log⁡(*n*) + *nmpg*), where *n* is the number of samples, *m* is the dimension of the data sets, *p* represents the population size, and *g* is the number of generations. In order for the algorithm to be effective the number of generations and the population size must be quite large. In addition like all wrappers, randomised algorithms take up more CPU time and more memory to run.

### 2.3. Embedded Techniques

Embedded techniques tend to do better computationally than wrappers but they make classifier dependent selections that might not work with any other classifier. That is because the optimal set of genes is built when the classifier is constructed and the selection is affected by the hypotheses the classifier makes. A well-known embedded technique is random forests. A random forest is a collection of classifiers. New random forests are created iteratively by discarding a small fraction of genes that have the lowest importance [41]. The forest with the smallest amount of features and the lowest error is selected to be the feature subset. A method called* block diagonal linear discriminant analysis* (BDLDA) [42] assumes that only a small number of genes are associated with a disease and therefore only a small number are needed in order for the classification to be accurate. To limit the number of features it imposes a block diagonal structure on the covariance matrix. In addition SVMs can be used for both feature selection and classification. Features that do not contribute to classification are eliminated in each round until no further improvement in the classification can be achieved [43].* Support vector machines-recursive feature elimination* (SVM-RFE) starts with all the features and gradually excludes the ones that do not identify separating samples in different classes. A feature is considered useful based on its weight resulting from training SVMs with the current set of features. In order to increase the likelihood that only the “best” features are selected, feature elimination progresses gradually and includes cross-validation steps [26, 44–46]. A major advantage of SVM-RFE is that it can select high-quality feature subsets for a particular classifier. It is however computationally expensive since it goes through all features one by one and it does not take into account any correlation the features might have [30]. SVM-RFE was compared against two wrappers: leave-one-out calculation sequential forward selection and gradient-based-leave-one-out. All three of these methods have similar computational times when run against a Hepatocellular Carcinoma dataset (7129 genes and 60 samples). GLGS outperforms the others, with LOOCSFS and SVM-RFE having similar performance errors [27].

The most commonly used methods on microarray data analysis are shown in Table 2.

## 3. Feature Extraction in Microarray Cancer Data

**Figure 3:**
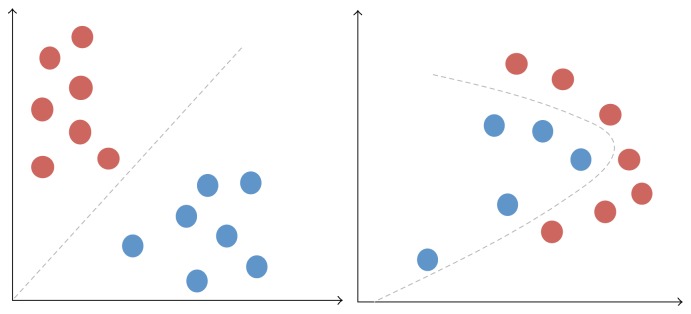
Linear versus nonlinear classification problems.

Early methods of machine learning applied to microarray data included simple clustering methods [47]. A widely used method was hierarchical clustering. Due to the flexibility of the clustering methods they became very popular among the biologists. As the technology advanced however the size of the data increased and a simple application of hierarchical clustering became too inefficient. The time complexity of hierarchical clustering is *O*(log⁡(*n* ^2^)), where *n* is the number of features. Biclustering followed hierarchical clustering as a way of simultaneously clustering both samples and features of a dataset leading to more meaningful clusters. It was shown that biclustering performs better than hierarchical clustering when it comes to microarray data but it is still a computationally demanding method [48]. Many other methods have been implemented for extracting only the important information from the microarrays thus reducing their size. Feature extraction creates new variables as combinations of others to reduce the dimensionality of the selected features. There are two broad categories for feature extraction algorithms: linear and nonlinear. The difference between linear and nonlinear problems is shown is Figure 3.

### 3.1. Linear

**Figure 4:**
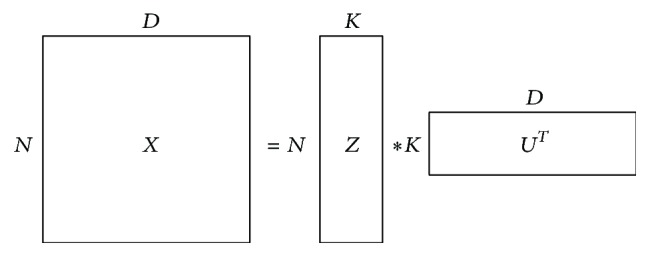
Dimensionality reduction using linear matrix factorization: projecting the data on a lower-dimensional linear subspace.

Linear feature extraction assumes that the data lies on a lower-dimensional linear subspace. It projects them on this subspace using matrix factorization. Given a dataset *X*: *N* × *D*, there exists a projection matrix *U*: *D* × *K* and a projection *Z*: *N* × *K*, where *Z* = *X* · *U*. Using *UU* ^*T*^ = *I* (orthogonal property of eigenvectors), we get *X* = *Z* · *U* ^*T*^. A graphical representation is shown in Figure 4.

The most well-known dimensionality reduction algorithm is* principal component analysis* (PCA). Using the covariance matrix and its eigenvalues and eigenvectors, PCA finds the “principal components” in the data which are uncorrelated eigenvectors each representing some proportion of variance in the data. PCA and many variations of it have been applied as a way of reducing the dimensionality of the data in cancer microarray data [58–64]. It has been argued [65, 66] that when computing the principal components (PCs) of a dataset there is no guarantee that the PCs will be related to the class variable. Therefore, supervised principal component analysis (SPCA) was proposed, which selects the PCs based on the class variables. They named this extra step the gene screening step. Even though the supervised version of PCA performs better than the unsupervised, PCA has an important limitation: it cannot capture nonlinear relationships that often exist in data, especially in complex biological systems. SPCA works as follows:

1. Compute the relation measure between each gene with outcome using linear, logistic, or proportional hazards models.
2. Select genes most associated with the outcome using cross-validation of the models in step (1).
3. Estimate principal component scores using only the selected genes.
4. Fit regression with outcome using model in step (1).

The method was highly effective in identifying important genes and in cross-validation tests was only outperformed by gene shaving, a statistical method for clustering, similar to hierarchical clustering. The main difference is that the genes can be part of more than one cluster. The term “shaving” comes from the removal or shaving of a percentage of the genes (normally 10%) that have the smallest absolute inner product with the leading principal component [67].

A similar linear approach is classical multidimensional scaling (classical MDS) or Principal Coordinates Analysis [68] which calculates the matrix of dissimilarities for any given matrix input. It was used for large genomic datasets because it is efficient in combination with Vector Quantization or *K*-Means [69] which assigns each observation to a class, out of a total of *K* classes [70].

### 3.2. Nonlinear

**Figure 5:**
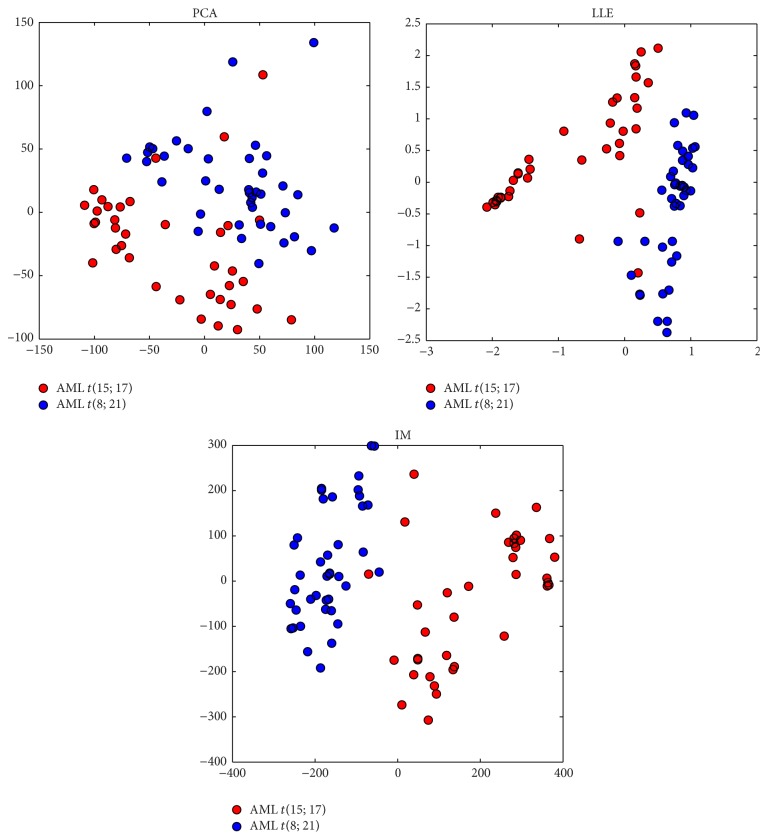
Visualisation of a Leukaemia dataset with PCA, manifold LLE, and manifold Isomap [34].

Nonlinear dimensionality reduction works in different ways. For example, a low-dimensional surface can be mapped on a high-dimensional space so that a nonlinear relationship among the features can be found. In theory, a lifting function *f*(*x*) can be used to map the features onto a higher-dimensional space. On a higher space the relationship among the features can be viewed as linear and therefore is easily detected. This is then mapped back on the lower-dimensional space and the relationship can be viewed as nonlinear. In practice kernel functions can be designed to create the same effect without the need to explicitly compute the lifting function. Another approach to nonlinear dimensionality reduction is by using manifolds. It is based on the assumption that the data (genes of interest) lie on an embedded nonlinear manifold which has lower dimension than the raw data space and lies within it. Several algorithms exist working in the manifold space and applied to microarrays. A commonly used method of finding an appropriate manifold, Isomap [71], constructs the manifold by joining each point only to its nearest neighbours. Distances between points are then taken as geodesic distances on the resulting graph. Many variants of Isomap have been used; for example, Balasubramanian and Schwartz proposed a tree connected version which differs in the way the neighbourhood graph is constructed [72]. The *k*-nearest points are found by constructing a minimum spanning tree using an *ϵ*-radius hypersphere. This method aims to overcome the drawbacks expressed by Orsenigo and Vercellis [73] regarding the robustness of the Isomap algorithm when it comes to noise and outliers. These could cause potential problems with the neighbouring graph, especially when the graph is not fully connected. Isomap has been applied on microarray data with some very good results [73, 74]. Compared to PCA, Isomap was able to extract more structural information about the data. In addition, other manifold algorithms have been used with microarray data such as* Locally Linear Embedding* (LLE) [75] and* Laplacian Eigenmaps* [76, 77]. PCA and similar manifold methods are used also for data visualisation as shown in Figure 5. Clusters can often be better separated using manifold LLE and Isomap but PCA is far faster than the other two.

Another nonlinear method for classification is* Kernel PCA*. It has been widely used [78, 79] since dimensionality reduction helps with the interpretability of the results. It does have an important limitation in terms of space complexity since it stores all the dot products of the training set and therefore the size of the matrix increases quadratically with the number of data points [80].

Neural methods can also be used for dimensionality reduction like* Self Organizing Maps* [81] (SOMs) or Kohonen maps that create a lower-dimensional mapping of an input by preserving its topological characteristics. They are composed of nodes or neurons and each node is associated with its own weight vector. SOMs training is considered to be “competitive” since when a training example is fed to the network its Euclidean distance with all nodes is calculated and it is assigned to that node with the smallest distance (Best Matching Unit (BMU)). The weight of that node along with its neighbouring nodes is adjusted to match the input. Another neural networks method for dimensionality reduction (and dimensionality expansion) uses* autoencoders*. Autoencoders are feed-forward neural networks which are trained to approximate a function by which data can be classified. For every training input the difference between the input and the output is measured (using square error) and it is back-propagated through the neural network to perform the weight updates to the different layers. In a paper that compares stacked autoencoders with PCA with Gaussian SVM on 13 gene expression datasets, it was shown that autoencoders perform better on the majority of datasets [82]. Autoencoders use fine-tuning, a back-propagation method for adjusting their parameters. Without back-propagation the autoencoders get very low accuracies. A general problem with the stacked autoencoders method is that a large number of internal layers can easily “memorise” the training data and create a model with zero error which will overfit the data and so be unable to classify future test data. SOMs have been used as a method of dimensionality reduction for gene expression data [77, 83] but it was never broadly adopted for analysis because it needs just the right amount of data to perform well. Insufficient or extraneous data can cause randomness to the clusters. Independent component analysis is also widely used in microarrays [84, 85] in combination with a clustering method.

*Independent Components Analysis* (ICA) finds the correlation among the data and decorrelates the data by maximizing or minimizing the contrast information. This is called “whitening.” The whitened matrix is then rotated to minimise the Gaussianity of the projection and in effect retrieve statistically independent data. It can be applied in combination with PCA. It is said that ICA works better if the data has been preprocessed with PCA [86]. This could merely be due to the decrease in computational load caused by the high dimension.

The advantages and disadvantages of feature extraction and feature selection are shown in Table 3 and in (5).

*Feature Selection and Feature Extraction: Difference between Feature Selection (Top) and Feature Extraction (Bottom)*. Consider

(5) X1X2⋮XN−1XNXi⋮XkXnX1X2⋮XN−1XNY1⋮YK=fX1X2⋮XN−1XN.

## 4. Prior Knowledge

Prior knowledge has previously been used in microarray studies with the objective of improving the classification accuracy. One early method for adding prior knowledge in a machine learning algorithm was introduced by Segal et al. [87]. It first partitions the variables into modules, which are gene sets that have the same statistical behaviour (share the same parents in a probabilistic network), and then uses this information to learn patterns. The modules were constructed using Bayesian networks and a Bayesian scoring function to decide how well a variable fits in a module. The parents for each module were restricted to only some hundreds of possible genes since those genes were most likely to play a regulatory role for the other genes. To learn the module networks Regression Trees were used. The gene expression data were taken from yeast in order to investigate how it responds to different stress conditions. The results were then verified using the Saccharomyces Genome Database. Adding prior knowledge reduces the complexity of the model and the number of parameters making analysis easier. A disadvantage however of this method is that it relies only on gene expression data, which is noisy. Many sources of external biological information are available and can be integrated with machine learning and/or dimensionality reduction methods. This will help overcoming one of the limitations of machine learning classification methods which is that they do not provide the necessary biological connection with the output. Adding external information in microarray data can give an insight on the functional annotation of the genes and the role they play in a disease, such as cancer.

### 4.1. Gene Ontology

Gene Ontology (GO) terms are a popular source of prior knowledge since they describe known functions of genes. Protein information found in the genes' GO indices has been combined with their expressions in order to identify more meaningful relationships among the genes [88]. A study infused GO information in a dissimilarity matrix [89] using Lin's similarity measure [90]. GO terms were also used as a way of weighting the longest partial path shared by two genes [91]. This was used with expression data in order to produce clusters using a pairwise similarity matrix of gene expressions and the weight of the GO paths. GO terms information integrated with gene expression was used by Chen and Wang [92], similar genes were clustered together, and SPCA was used to find the PCs. GO terms have been used to derive information about the biological similarity of a pair of genes. This similarity was used as a modified distance metric for clustering [93]. Using a similar idea in a later publication, similarity measures were used to assign prior probabilities for genes to belong in specific clusters [94] using an expectation maximisation model. Not all of these methods have been compared to other forms of dimensionality reduction such as PCA or manifold which is a serious limitation as to their actual performance. It is however the case that in all of those papers an important problem regarding GO terms is described. Some genes do not belong in a functional group and therefore cannot be used. Additionally GO terms tend to be very general when it comes to the functional categories and this leads to bigger gene clusters that are not necessarily relevant in microarray experiments.

### 4.2. Protein-Protein Interaction

Other studies have used protein-protein interaction (PPI) networks for the same purpose [95]. Subnetworks are identified using PPI information. Iteratively more interactions are added to each subnetwork and scored using mutual information between the expression information and the class label in order to find the most significant subnetwork. The initial study showed that there is potential for using PPI networks but there is a lot of work to be done. Prior knowledge methods tend to use prior knowledge in order to filter data out or even penalise features. These features are called outliers and normally are the ones that vary from the average. The Statistical-Algorithmic Method for Bicluster Analysis (SAMBA) algorithm [96] is a biclustering framework that combines PPI and DNA binding information. It identifies subsets that jointly respond in a subset of conditions. It creates a bipartite graph that corresponds to genes and conditions. A probabilistic model is created based on weights assigned on the significant biclusters. The results for lymphoma microarray showed that the clusters produced were highly relevant to the disease. A positive feature of the SAMBA algorithms is that it can detect overlapping subsets but it has important limitations in the weighting process. All sources are assigned equal weights and they are not penalised according to their importance or reliability of the source.

### 4.3. Gene Pathways

The most promising results were shown when using pathway information as prior knowledge. Many databases containing information on networks of molecular interaction in different organisms exist (KEGG, Pathway Interaction Database, Reactome, etc.). It is widely believed that these lower level interactions can be seen as the building blocks of genetic systems and can be used to understand high-level functions of the biological systems. KEGG pathways have been quite popular in network constrained methods which use networks to identify gene relations to diseases. Not many methods used pathway knowledge but most of them treat pathways as networks with directed edges. A network-based penalty function for variable selection has been introduced [97]. The framework used penalised regression, after imposing a smoothness assumption on the regression coefficients based on their location on the gene network. The biological motivation of this penalty is that the genes that are linked on the networks are expected to have similar functions and therefore bigger coefficients. The weights are also penalised using the sum of squares of the scaled difference of the coefficients between neighbour vertices in the network in order to smooth the regression coefficients. The results were promising in terms of identifying networks and subnetworks of genes that are responsible for a disease. However the authors only used 33 networks and not the entire set of available networks. A similar approach also exists. It is theoretical model which according to the authors can be applied to cancer microarray data but to date has not been explored [98]. The proposed method was based on Fourier transformation and spectral graph analysis. The gene expression profiles were reconstructed using prior knowledge to modify the distance from gene networks. They use the assumption that the information lies in the low frequency component of the expression while the high frequency component is mostly noise. Using spectral decomposition the smaller eigenvalues and corresponding eigenvectors are kept (the smaller the eigenvalue the smoother the graph). A linear classifier can be inferred by penalising the regression coefficients based on network information. The biological Pathway-Based Feature Selection (BPFS) algorithm [99] also utilizes pathway information for microarray classification. It uses SVMs to calculate the marginal classification power of the genes and puts those genes in a separate set. Then the influence factor for each of the genes in the second set is calculated. This is an indication of the interaction of every gene in the second set with the already selected genes. If the influence factor is low the genes are added to the set of the selected genes. The influence factor is the sum of the shortest pathway distances that connect the gene to be added with each other gene in the set.

## 5. Summary

This paper has presented different ways of reducing the dimensionality of high-dimensional microarray cancer data. The increase in the amount of data to be analysed has made dimensionality reduction methods essential in order to get meaningful results. Different feature selection and feature extraction methods were described and compared. Their advantages and disadvantages were also discussed. In addition we presented several methods that incorporate prior knowledge from various biological sources which is a way of increasing the accuracy and reducing the computational complexity of existing methods.

## Figures and Tables

**Algorithm 1:**
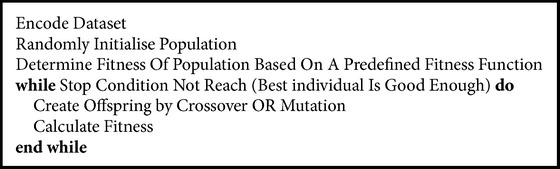
Genetic algorithm.

**Algorithm 2:**
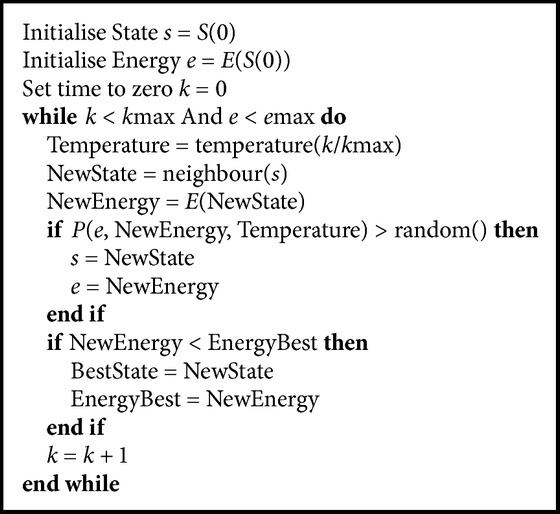
Simulated annealing algorithm.

**Table 1:** Deterministic versus randomised wrappers.

Deterministic	Randomised
Small overfitting risk	High overfitting risk
Prone to local optima	Less prone to local optima
Classifier dependent	Classifier dependent
—	Computationally intensive

Comparison between deterministic and randomised wrappers.

**Table 2:** Feature selection methods applied on microarray data.

Method	Type	Supervised	Linear	Description
*t*-test feature selection [49]	Filter	—	Yes	It finds features with a maximal difference of mean value between groups and a minimal variability within each group

Correlation-based feature selection (CFS) [50]	Filter	—	Yes	It finds features that are highly correlated with the class but are uncorrelated with each other

Bayesian networks [51, 52]	Filter	Yes	No	They determine the causal relationships among features and remove the ones that do not have any causal relationship with the class

Information gain (IG) [53]	Filter	No	Yes	It measures how common a feature is in a class compared to all other classes

Genetic algorithms (GA) [33, 54]	Wrapper	Yes	No	They find the smaller set of features for which the optimization criterion (classification accuracy) does not deteriorate

Sequential search [55]	Wrapper	—	—	Heuristic base search algorithm that finds the features with the highest criterion value (classification accuracy) by adding one new feature to the set every time

SVM method of recursive feature elimination (RFE) [30]	Embedded	Yes	Yes	It constructs the SVM classifier and eliminates the features based on their “weight” when constructing the classifier

Random forests [41, 56]	Embedded	Yes	Yes	They create a number of decision trees using different samples of the original data and use different averaging algorithms to improve accuracy

Least absolute shrinkage and selection operator (LASSO) [57]	Embedded	Yes	Yes	It constructs a linear model that sets many of the feature coefficients to zero and uses the nonzero ones as the selected features.

Different feature selection methods and their characteristics.

**Table 3:** Advantages and disadvantages between feature selection and feature extraction.

Method	Advantages	Disadvantages
Selection	Preserving data characteristics for interpretability	Discriminative power
		Lower shorter training times
		Reducing overfitting

Extraction	Higher discriminating power	Loss of data interpretability
	Control overfitting when it is unsupervised	Transformation maybe expensive

A comparison between feature selection and feature extraction methods.
